# Cigarette Smoke-Induced Epithelial-to-Mesenchymal Transition: Insights into Cellular Mechanisms and Signaling Pathways

**DOI:** 10.3390/cells13171453

**Published:** 2024-08-29

**Authors:** Sarah Mohammed Alqithami, Amrita Machwe, David K. Orren

**Affiliations:** Department of Toxicology and Cancer Biology, University of Kentucky College of Medicine, Lexington, KY 40536, USA

**Keywords:** cigarette smoke, epithelial-to-mesenchymal transition, EMT, normal human bronchial cell, 2D cell cultures

## Abstract

This review delves into the molecular complexities underpinning the epithelial-to-mesenchymal transition (EMT) induced by cigarette smoke (CS) in human bronchial epithelial cells (HBECs). The complex interplay of pathways, including those related to WNT//β-catenin, TGF-β/SMAD, hypoxia, oxidative stress, PI3K/Akt, and NF-κB, plays a central role in mediating this transition. While these findings significantly broaden our understanding of CS-induced EMT, the research reviewed herein leans heavily on 2D cell cultures, highlighting a research gap. Furthermore, the review identifies a stark omission of genetic and epigenetic factors in recent studies. Despite these shortcomings, the findings furnish a consolidated foundation not only for the academic community but also for the broader scientific and industrial sectors, including large tobacco companies and manufacturers of related products, both highlighting areas of current understanding and identifying areas for deeper exploration. The synthesis herein aims to propel further research, hoping to unravel the complexities of the EMT in the context of CS exposure. This review not only expands our understanding of CS-induced EMT but also reveals critical limitations in current methodologies, primarily the reliance on 2D cell cultures, which may not adequately simulate more complex biological interactions. Additionally, it highlights a significant gap in the literature concerning the genetic and epigenetic factors involved in CS-induced EMT, suggesting an urgent need for comprehensive studies that incorporate these types of experiments.

## 1. Introduction

Cigarette smoke (CS) has been strongly correlated with at least 80% of lung cancer cases [1], encompassing both small cell lung cancer and non-small cell lung cancer [2]. In the United States alone, smoking stands as a primary contributor to lung cancer, which ranked highest in terms of cancer incidence and mortality for both men and women in the year of 2023 [3]. Additionally, smoking is associated with cancer in various other organs, including the mouth, pharynx, larynx, esophagus, stomach, pancreas, cervix, kidney, and bladder (see Table 1) [4]. Moreover, CS can contribute to the development of second primary tumors [5,6], promote cancer metastasis [7,8], and contribute to the onset of other lung diseases [9,10] such as chronic obstructive pulmonary disease (COPD) including both emphysema and chronic bronchitis, pulmonary fibrosis, and asthma [11,12]. The findings of comparative studies between smokers and nonsmokers have demonstrated that smokers experience an accelerated decline in lung function [13,14,15] and are at a higher risk of developing both lung cancer and COPD simultaneously [16,17].

In 2020, the global impact of smoking was significant, affecting approximately 1.18 billion individuals worldwide [18]. Tobacco smoking resulted in the deaths of 7 million people during that year [18], and the World Health Organization (WHO) projects that it is anticipated to cause the deaths of 8 million individuals annually [19]. Nevertheless, despite these alarming figures, the projected worldwide reduction in tobacco use is predicted to reach just 24% by 2025, undershooting the WHO’s 30% target given current tobacco control efforts.

CS-induced pathogenicity is driven by several factors, including inflammatory responses, oxidative stress [20], genomic instability [21], and the epithelial-to-mesenchymal transition (EMT). The EMT has been associated with structural changes in the airways, leading to remodeling lung diseases [22,23]. Extensive research has demonstrated that the EMT is an active process in the airways of both smokers and smokers with COPD, contributing to both fibrosis and malignant transformation [24,25,26,27]. Further, a relationship between the EMT and cancer stem cells is supported by research indicating that the EMT is associated with the acquisition of stemness in various cancer types, such as breast, lung, colon, prostate, and ovarian cancers [5,28,29]. The EMT not only helps in the transportation of cancer cells, but also with the increase in their proliferative capability, which aids in the sustenance and growth of the tumor mass. By the same token, the EMT has been credited for the conversion of early carcinoma to invasive cancer as well as for maintaining the stemness of cancer cells [30,31,32].

CS-induced EMT is associated with emanating pathways like ERK5, MAPK, and Src that are known for their role in orchestrating the EMT and cancer progression. Also, through these pathways, other transcription factors such as AP- 1 and TWIST are activated to promote the EMT and cancer metastasis [33]. Additionally, it has been observed that on CS exposure, the EMT is promoted by the induction of factors such as hypoxia-inducible factor 1-alpha (HIF-1α) and transforming growth factor β1 (TGF-β1) [8,34]. Another approach mentioned in several works is the issue of defining exact molecules that may be used for CS antagonism and prevention of the development of the EMT. For example, molecules such as FERMT3, REV-ERBα, and TRPV4 have been targeted for the purpose of inhibiting the EMT and increasing the anti-inflammatory response of CS exposure [35]. Further, it has also been established that natural compounds such as resveratrol and piperine exert protective roles against CS-triggered EMT through regulating core molecular signaling pathways [3,36].

Our lab has observed a morphological effect upon treatment of human bronchial epithelial cell lines with CS, which we believe to be at least EMT-like. Thus, we wanted to explore the literature on the topic of CS-induced EMT. Although existing reviews have explored the influence of cigarette smoking on the EMT in COPD [37] and lung cancer [38], there is a notable gap in comprehensive reviews specifically examining the promotion of the EMT in normal bronchial cells by CS. Thus, the objective of this literature review is to fill this gap by investigating the involvement of the EMT in the pathology induced by CS in human bronchial epithelial cells and unraveling the underlying mechanisms. Gaining a deeper understanding of these mechanisms holds significant implications for the prevention of CS-induced lung diseases and the identification of potential therapeutic targets. Table 1 summarizes the major findings and related to smoking and cancer prognosis.

## 2. Cigarette Smoke (CS) and Human Bronchial Epithelial Cells

CS negatively affects human bronchial epithelial cells. This review systematically identifies relevant studies that investigate the pathological interactions between CS and these cells.

### 2.1. Toxic Components of Cigarette Smoke

CS is a complex mixture containing over 9500 chemical compounds [39], with approximately 6010 identified in tobacco smoke [40]. Although all components of CS are harmful, recent research has placed particular emphasis on their carcinogenic effects [41,42,43]. In 2012, the U.S. Food and Drug Administration (FDA) compiled a list of 93 substances present in tobacco products, classifying 79 of them as carcinogens and 25 as respiratory toxicants [44]. An updated list by the International Agency for Research on Cancer [2] in 2022 revealed 83 carcinogenic compounds in tobacco, including 80 in the smoke [45]. More than 20 of these constituents have been identified as pulmonary carcinogens based on substantial evidence demonstrating their carcinogenic effects in either laboratory animals or humans. These compounds can be broadly classified into polycyclic aromatic hydrocarbons (PAHs), Aza-arenes, tobacco-specific nitrosamines (TSNAs), miscellaneous organic compounds, and inorganic compounds [46]. Similarly, certain carcinogens, such as the PAH benzo[α]pyrene and TSNAs like nicotine-derived nitrosamine ketone (NNK) and N-nitrosonornicotine (NNN), have been proposed for regulation by the WHO [47].

### 2.2. Human Bronchial Epithelial Cells

The human bronchial epithelium, which lines the inner surface of the bronchi, plays a crucial role in maintaining the integrity of the airway barrier [48]. Given its constant exposure to inhaled particles, including those present in CS, this epithelium is involved in various respiratory conditions associated with CS, such as asthma, COPD, and lung cancer. Due to this reason, researchers often employ human bronchial epithelial cell (HBEC) models to investigate the intricate cellular and molecular processes that occur in the airway epithelium upon exposure to CS and its association with respiratory conditions [49].

The HBEC models utilized in research encompass primary and immortalized normal human bronchial epithelial cells. Primary HBECs are derived from lung cancer and other lung diseases undergoing surgical resection, specifically from non-cancerous regions of the lung [50]. These primary cells are sourced from different donors, each with unique genetic backgrounds and distinct exposure histories to CS and other stimuli. Although primary HBECs closely mimic the physiological state of cells in the body, their culture lifespan is restricted and not long-lasting [50,51].

On the other hand, immortalized HBECs are primary HBECs that have been genetically modified to overcome their limited replicative lifespan. Immortalization methods involve introducing exogenous human telomerase reverse transcriptase (hTERT), viral oncoproteins (such as HPV-16 E6 and E7, or SV40 T-antigen), defective SV40 virus genomes, or additional cellular genes like cyclin-dependent kinase 4 (CDK4) to inhibit cell cycle checkpoints and prevent senescence [52,53]. These immortalized cell lines retain the characteristic phenotype and functionality of normal bronchial epithelial cells, including the epithelial morphology, expression of epithelial markers, ability to form tight junctions, and responsiveness to environmental stimuli. They are also described as being more like basal cells, which retain the capacity to repair the lung epithelium and, under appropriate conditions, can differentiate into the various cell types that line the airways [21,52].

## 3. Evidence of Cigarette Smoke-Induced EMT

In studies that investigate the effects of CS on cells, researchers commonly employ two different forms of CS: cigarette smoke extract (CSE, the gas phase of tobacco smoke) and cigarette smoke condensate (CSC, the particulate phase of tobacco smoke). The CSE method involves burning a cigarette with the assistance of a pump through a serum-free medium and passing the resulting solution through a sterile filtration process to remove bacteria; the final solution is considered 100% CSE [54,55,56]. On the other hand, the CSC method entails collecting the particulate phase of the smoke on a Cambridge filter pad after generating the smoke with a smoking machine. The absorbed material is dissolved in a solvent like dimethyl sulfoxide (DMSO), filtered to obtain a sterile solution, and then added to the cell culture [57,58]. Both CSE and CSC solutions can be diluted to achieve the desired concentrations for experimental purposes. It is important to note that researchers may employ variations on or modifications to these methods based on their specific experimental requirements. One alternative approach is testing the individual components of CS, such as nicotine or benzopyrene, by directly adding them to the cell culture [59,60].

CSC and CSE solutions display distinct components. The chemical constituents in the gas phase differ from those in the particulate phase. For example, aldehydes, benzene, and 1,3-butadiene are present at significantly higher levels in the gas phase, whereas constituents like nicotine, benzopyrene, and TSNAs are more prevalent in the particulate phase [61,62]. Interestingly, despite both solutions containing nicotine, the CSC solution exhibits significantly higher nicotine concentrations compared to that in CSE. Furthermore, the types of volatile organic compounds (VOCs) generated during the incomplete combustion of tobacco differ between CSE and CSC. CSE solution primarily consists of lighter VOCs with a molecular weight below 100 g/mol, whereas the CSC solution contains relatively heavier VOCs with a molecular weight exceeding 100 g/mol [63].

### 3.1. Cigarette Smoke (CS)-Induced Epithelial-to-Mesenchymal Transition (EMT)

The induction of EMT by CSE and CSC is influenced by both the duration of exposure and concentration of the CS. Research has demonstrated that the duration of CS exposure plays a significant role in triggering the EMT in HBECs. Some studies have observed a pronounced transition within 24 or 48 h of exposure [64,65,66], while others have found that more days of exposure are necessary to achieve a more significant and profound transition [67,68]. However, it is important to note that not all studies have observed this transition [54,69,70], indicating potential dissimilarities due to different experimental conditions. In studies that expose HBECs to CS for weeks or months, the epithelial cells exhibit cancer-like traits such as anchorage-independent growth, in addition to displaying characteristics associated with the EMT [71,72,73,74,75].

In addition to the duration of exposure, the dosage or concentration of CS used in treating HBECs is also a crucial factor. Researchers have conducted experiments utilizing various CS doses to ascertain the optimal dosage capable of inducing EMT characteristics without triggering cytotoxicity, and identified the spectrum of variable concentrations [65,66,76,77,78]. The dissimilarities among concentrations can be attributed to several factors. These include the genetic diversity and exposure histories of HBEC line donors, changes in cell line characteristics over time, variations in the preparation of CSC/CSE between labs, and variations in the specific bronchial regions studied (such as small vs. large airways). To achieve a clear understanding of the induction of the EMT by CS in normal HBECs, we have divided the available evidence into two types: structural and functional evidence.

### 3.2. Structural Evidence

**Figure 1:**
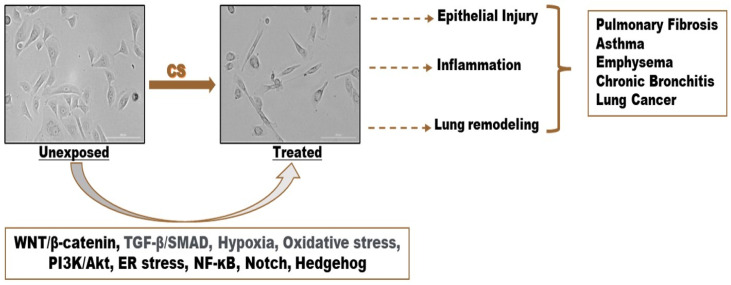
Immortalized HBECs after exposure to CS: potential pathological consequences and involved signaling pathways.

Exposure of HBECs to CS induces noticeable changes in cell morphology and organization, providing structural evidence of the EMT (Figure 1).

When exposed to CS, HBECs undergo a distinct alteration in their morphology, characterized by the loss of their typical cobblestone-like appearance and the acquisition of an elongated and mesenchymal-like shape [57,59,67,68,71,75,79]. This morphological shift is accompanied by a decrease in cell adhesion, disruption of cell–cell contacts, and compromised barrier integrity [74]. CS exposure leads to reduced expression levels of tight junction proteins and adherent junction proteins, such as cadherins and catenin family members, resulting in the disruption of adhesive interactions between neighboring epithelial cells. A study by Eurlings et al. demonstrated that CSE stimulation significantly decreased the adhesion of BEAS-2B cells on collagen I- and fibronectin-coated dishes [56]. Exposure to CSE, CSC, and nicotine also leads to the downregulation of extracellular matrix (ECM) proteins and the upregulation of proteins involved in ECM degradation [59,64,69]. Notably, CS-treated HBECs were found to produce higher levels of collagen in response to CSE exposure [56]. These structural changes observed after CS exposure are accompanied by the downregulation of epithelial markers and the upregulation of mesenchymal markers in a dose- and time-dependent manner, as presented in Table 1. These alterations are indicative of the transition from an epithelial to a mesenchymal phenotype and have been consistently observed in the lungs of smokers in several studies [24,27,80], confirming the association between the EMT and CS exposure.

### 3.3. Functional Evidence

CS-induced EMT is evidenced by different functional forms supported by a number of studies. For instance, due to the disruption of the intercellular junction and ECM upon exposure to CS, cells acquire migratory and invasive capacity, which is a hallmark of the EMT [58,67,68,81]. Similarly, in CS-induced EMT, cell proliferation increases [58,82,83]. Moreover, cell apoptosis is also affected by CS. Although the EMT is known to inhibit cell apoptosis, CS-induced EMT increases cell apoptosis [84]. Lastly, CS-treated cells increased the expression of the ferroptosis markers transferrin receptor (TfR), ferritin light-chain (FtL), and glutathione peroxidase 4 (GPX4) [85].

## 4. Molecular Mechanisms of CS-Induced EMT

**Figure 2:**
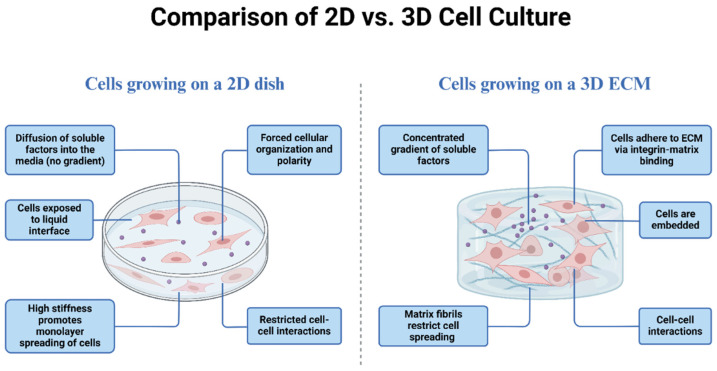
Comparative overview of cellular environments: 2D vs. 3D cultures in the study of EMT.

Different signaling pathways, like the NF-KB signaling pathway, endoplasmic reticulum stress, the PI3K/Akt pathway, the oxidative stress signaling pathway, the hypoxia signaling pathway, transforming growth factor β (TGF-β), and the WNT signaling pathway, that induce CS-induced EMT have been discovered in normal HBECs (Figure 2). Moreover, these signaling pathways share common characteristics in both normal cells and diseases like lung cancer and COPD. The inhibition of the EMT is a promising strategy for addressing cancer metastasis, pulmonary fibrosis, and smoker-related inflammation [86]. The EMT, crucial in these conditions, involves the transformation of epithelial cells into mesenchymal cells, enhancing cell migration and invasion. Targeting key EMT regulators and pathways may lead to new treatments for these challenging pathologies [87]. The EMT in normal bronchial cells, triggered by exposure to CS, is believed to be a critical factor contributing to the development of pathologies in later stages [24,38,82]. The following sections discuss the signaling pathways that have been identified in previous studies.

### 4.1. WNT/β-Catenin Signaling Pathway

The canonical wingless-related integration site (WNT) signaling pathway is a β-catenin-dependent pathway in the EMT [88,89,90]. In this case, WNT ligands bind to the frizzled/LRP receptor, which then leads to cytosolic stabilization and nuclear translocation of β-catenin. β-catenin acts as a powerful transactivator of T-cell factor/lymphoid enhancer-binding factor (TCF/LEF) transcription factors, which regulate the transcription of various remodeling genes [91].

The activation of the WNT/β-catenin pathway in response to CS exposure has been widely observed in both in vitro *and* in vivo studies. Studies have shown the elevated expression of WNT ligands and upregulation of the pathway in the lung tissue of smokers, as well as in normal HBECs upon exposure to CS. Exposure to CSE or nicotine for 6 h or 72 h, respectively, resulted in the upregulation of WNT-5B mRNA in both 16HBE and BEAS-2B cells [59,64,92]. Furthermore, 16HBE14o cells treated with 1% CSE every other day for 8 days exhibited the increased expression of key genes involved in the WNT/β-catenin pathway, such as Wnt family member 3 (WNT3), dishevelled family protein 3 (DLV3), axis inhibition (AXIN), and β-catenin; similar genes were upregulated in smokers’ lungs [93]. Moreover, the pathway antagonists were downregulated upon exposure of immortalized HBEC to 1% CSC [72].

The main component of the WNT/β-catenin pathway, β-catenin, demonstrated increased expression and translocation into the nucleus following CS exposure. Two studies revealed that after a 24h treatment of HBEC with nicotine and BEAS-2 with CSC, there was a notable increase in β-catenin levels, along with its localization in the cell nucleus [59,69]. The upregulation of this pathway, along with the activation of β-catenin, facilitates its interaction with transcription factors (TCF/LEF). This interaction is crucial for initiating the transcription of EMT remodeling genes such as fibronectin, matrix metalloproteinase 2 and 9 (MMP-2 and MMP-9), and Snail in normal HBECs [64]. Zou et al. found that nicotine increased the expression of WNT-3a in primary HBECs, leading to subsequent upregulation of α-smooth muscle actin (α-SMA), vimentin, MMP-9, and type I collagen expression after 72 h of exposure. Simultaneously, the expression of E-cadherin, an epithelial marker, was downregulated, confirming the involvement of the Wnt3a/β-catenin signaling pathway in CS-induced EMT [59].

Apart from the canonical WNT signaling pathway, exposure to CS could also mediate the EMT in HBECs by activating the non-canonical WNT signaling pathway. A study found that after treating BEAS-2B with 10% CSE, the expression of WNT-5B increased, which subsequently increased phosphorylation of the non-canonical signaling molecule p38. This indicates that CSE causes WNT-5B to become dysregulated, which in turn leads to airway remodeling in COPD [64].

### 4.2. TGF-β/SMAD Signaling Pathway

Studies investigating the EMT have often employed transforming growth factor-β (TGF-β) stimulation, which is associated with tissue remodeling and fibrosis [94,95]. The TGF-β ligands activate receptor complexes involving type II and type I receptor serine/threonine kinases, leading to the activation of SMAD and alternative signaling pathways. SMADs, including R-SMADs (SMAD1, -2, -3, -5, and -8) and Co-SMAD (SMAD4), play crucial roles as intracellular transcriptional effectors of TGF-β family receptor signaling. Inhibitory SMADs (I-SMADs), such as SMAD6 and SMAD7, function as negative signal regulators, counteracting canonical SMAD signaling [96].

CS increases TGF-β1 production and induces the EMT in HBECs. Exposing HBECs to nicotine caused an increase in the total amounts of TGF-β1 production at 12, 24, and 72 h [59]. Furthermore, exposing BEAS-2B cells to 10% CSE for 48 h showed an upregulation of TGF-β1/SMAD pathway components, including TGF-β1, TGF-βR1, phospho-SMAD2, and phospho-SMAD3 [77]. A study by Mahmood et al. demonstrated that p-SMAD2/3 expression was associated with smoking and found a significant correlation between SMAD2/3 expression and EMT activity markers [97]. 16HBE cells treated with nicotine or 5% CSE for 72 h showed an upregulation of mesenchymal markers through the phosphorylation of SMAD2/3 and similar findings in BEAS-2B exposed to 1% CSE for 24 h [76,81,98,99]. A study exposing BEAS-2B to 10% CSE for 24 h showed the upregulation of fibronectin, an EMT remodeling gene, through activating downstream SMAD3 and p38, which are both downstream targets of TGF-β signaling, and using inhibitors of these targets or for the receptor showed the abrogation of EMT markers [64]. Treating 16HBE with 3% CSE for 48 h upregulated the protein expression levels of TGF-β1, p-SMAD3, and SMAD3 in a dose-dependent manner, which subsequently induced the EMT, indicating the involvement of the TGF-β1/SMAD3 signaling pathway in CS-induced EMT [100].

Interestingly, a study by Heijink et al. revealed potential crosstalk between WNT signaling and SMAD signaling. WNT-5B was shown to activate SMAD3 and upregulate the epithelial expression of downstream targets like fibronectin, MMP-2, MMP-9, and Snail via TGF-β/SMAD3-dependent signaling. This indicates a complex interplay between WNT and TGF-β signaling pathways in the context of the EMT induced by CS [64].

In addition, WNT signaling can increase the de novo synthesis of TGF-β, a key inducer of CS-associated EMT. TGF-β is initially produced and secreted in a latent form, incorporated into the ECM. During tissue damage, various mechanisms can convert latent TGF-β into its active form, enabling it to bind to its receptor and regulate repair and remodeling processes, including ECM changes and proteolytic activation via plasmin and MMP-2/9 [24]. Studies have demonstrated that in HBECs exposed to nicotine, there is an upsurge in TGF-β1 expression induced by WNT3a, leading to the EMT. Furthermore, inhibiting TGF-β1 in these cells partially counteracted nicotine-induced EMT, highlighting the interaction between TGF-β1 and the WNT pathway during this process. Similar findings were observed with WNT-5B, reinforcing the importance of the WNT-TGF-β crosstalk in CS-induced EMT [59,64].

### 4.3. Hypoxia Signaling Pathway

Hypoxia signaling is another mechanism that could underlie the EMT in response to CSE in normal bronchial cells. In expression studies, upon the exposure of BEAS-2B cells to CSE, there was a notable increase in the mRNA expression of carbonic anhydrase 9 (CA9), a gene responsive to hypoxia-inducible factor 1 (HIF1). Knocking down HIF1α resulted in a reduction in mesenchymal markers, including plasminogen activator inhibitor-1 (PAI1), vimentin, and fibronectin, in response to CS, indicating that CSE-induced EMT may occur through HIF1α activation [56]. Additionally, the treatment of BEAS-2B cells with side-stream smoke led to the induction of HIF1α expression, further supporting the potential role of hypoxia signaling in the EMT process triggered by CS [101].

### 4.4. Oxidative Stress Signaling Pathway

CS is rich in free radicals, leading to the generation of reactive oxygen species [102], which cause oxidative damage in bronchial epithelial cells. Exposure to CS upregulates the level of oxidative stress, which is the direct mechanism that causes injury to cells and tissues [52]. The intracellular ROS level and the concentration of H_2_O_2_ in culture medium increased evidently after CSE treatment and the expression levels of antioxidative genes like CD147, α-SMA, and Vimentin were also upregulated after CSE exposure in HBE cells [55]. In a study conducted by Guan et al., the significant contribution of oxidative stress to the process of CS-induced EMT in HBECs was validated. This was confirmed by using Ginsenoside Rg1, the primary component of a traditional herbal medicine known for its antioxidant properties [77]. Likewise, in a study by Zhou et al., pretreatment of HBECs with N-acetylcysteine (NAC), an antioxidant, led to a reduction in the expression of upregulated EMT markers caused by CS exposure. Simultaneously, the antioxidant treatment increased the expression of epithelial markers compared to their levels before antioxidant intervention [55].

Similarly, Liu et al. demonstrated similar findings, showing that exposing HBECs to CSE resulted in the upregulation of oxidative stress markers, including ROS and malondialdehyde (MDA) levels, while suppressing superoxide dismutase (SOD) levels and inducing EMT markers. However, upon adding desferoxamine, a compound that reduces oxidative stress, the ROS and MDA levels were decreased, and the SOD levels were elevated, alongside a modulation of EMT protein markers [85]. Similarly, 16HBE cells exposed to CSE showed comparable results [98]. These findings collectively emphasize the critical role of oxidative stress as a key mediator of the EMT in response to CS exposure.

### 4.5. PI3K/Akt Pathway

The involvement of the phosphatidylinositol 3-kinase (PI3K)-Akt pathway in the EMT is well established [103]. It plays a crucial role in CS-induced EMT in human type II alveolar epithelial cells [104]. The activation of Akt, the downstream effector of PI3K, has been found to induce the transcription factor Snail, which in turn represses the expression of the E-cadherin gene [104].

Zhang et al. demonstrated that exposure to CSE led to time-dependent activation of Akt phosphorylation in HBECs, suggesting a direct impact of Akt on epithelial cell morphology, motility, and invasiveness. The PI3K/Akt pathway is also implicated in the molecular and morphological changes observed in BEAS-2B cells after CSE treatment [105]. Similarly, treating BEAS-2B cells with 5% CSE for 5 days elevated the level of phosphorylated Akt (p-Akt) proteins. Antagonizing PI3K in these cells inhibited the development of CSE-induced EMT by reversing the reduction in E-cadherin and the elevation in vimentin expression [68]. In addition, Jiang et al. demonstrated that using an AKT inhibitor for 30 min prior to CSE exposure significantly downregulated the mRNA expression of mesenchymal markers in these cells [98]. Collectively, these studies highlight the significance of the PI3K-Akt pathway in mediating the EMT in response to CS.

### 4.6. Endoplasmic Reticulum (ER) Stress

ER stress has been found to occur in lung epithelial cells, especially in the bronchial epithelial cells of smokers, which is induced by CS [106,107,108]. In the ER, glucose-regulated protein 78 (GRP78) binds to three transmembrane sensor proteins, inositol requiring enzyme 1 (IRE1), activating transcription factor-6 (ATF6), and PKR-like ER kinase (PERK), maintaining each in its inactive state. During ER stress, GRP78 is released from IRE1, ATF6, and PERK, so these three transmembrane sensor proteins can assume their activated state. p-IRE1 splices the mRNA of x-box binding protein 1 (XBP1) into the mature form sec-XBP1, which can activate a series of genes involved in ER-associated protein degradation or protein folding, thus playing a protective role in ER stress. However, the downstream effects of the phosphorylation of IRE1 include the activation of c-Jun N-terminal kinase (JNK), which mediates some harmful effects such as proliferation, differentiation, carcinogenesis, or apoptosis [81].

Lin et al. found that nicotine increased the protein level of the ER stress marker in HBECs in a time-dependent manner [80]. Nicotine is also found to induce ER stress by increasing the expression of ER stress markers such as p-IRE1, sec-XBP1, and GRP78 in 16HBECs [81]. This indicated the role of CS in endoplasmic reticulum stress.

### 4.7. NF-KB Signaling Pathway

The NF-κB family comprises various proteins, such as p65 (RelA), p50, c-Rel, and RelB. These proteins can combine to form heterodimers or homodimers, with the most prevalent form being the p65/p50 heterodimer [109]. Evidence suggests an in vitro correlation between the EMT and the enhanced expression and activation of NF-κB [110,111]. Kumar et al. demonstrated that NF-κB plays a transcriptional role in upregulating the induction of master-switch transcription factors crucial for the EMT, including TWIST1, SNAI2, and ZEB2 [112]. In a separate study, Zhao et al. found that prolonged exposure to CSE resulted in the increased expression of phosphorylated p65 (p-p65) in HBECs. Additionally, the localization of p65 changed to the nuclear region upon stimulation with CS. Moreover, the activation of the NF-κB pathway following CS exposure led to an upregulation of certain EMT markers [113]. These findings suggest the role of CS in the EMT through the NF-KB signaling pathway.

### 4.8. Notch Signaling Pathway

The Notch signaling pathway plays a significant role in determining cell fate decisions, mainly during the regulation of the EMT process and the conservation of cancer stem cells. TNF receptor superfamily members, such as jagged and delta-like, activate the Notch intracellular domain (NICD) by releasing it into the nucleus, where it can interact with the transcription factors required to control genes related to cell differentiation, proliferation, and survival [114]. Concerning the relationship with CS exposure, Notch signaling has been reported to facilitate the EMT by increasing the expression of mesenchymal markers and decreasing that of epithelial markers [115]. Many studies using bronchial epithelial cells exposed to CS have demonstrated that the activation of Notch initiates the translation of EMT-related transcription factors such as Snail and Slug and inhibits the translation and expression of E-cadherin [102,114].

The Notch pathway seems to be a therapeutic target to suppress the EMT and lessen CSC stemness properties in the context of lung cancer. The enhancement of the interaction between Notch signaling and other pathways such as WNT and TGF-β/SMAD equally supports the EMT, which is responsible for the stemness and invasive ability of cancer stem cells. Notch signaling is not only essential for developmental problems but is also implicated in other diseases, such as tumorigenesis [7,35,116].

### 4.9. Hedgehog Signaling Pathway

Another very important pathway is the Hedgehog (Hh) signaling pathway, which is also involved with the regulation of the EMT and cancer stem cells, and it is known that dysregulation of this signaling pathway supports tumorigenesis, the promotion of the EMT, and the preservation of cancer stem cells [117]. Components of Hh signals have been shown to be upregulated by CS, which in turn activates transcription factors like GLI family zinc finger 1 (GLI1), which enhances mesenchymal features and downregulates epithelial features, thereby enhancing the EMT process. Moreover, Hh signaling is essential for the perpetual proliferation and the maintenance of the cellular stem qualities of cancer stem cells and, more specifically, in lung cancer associated with smoking. Antagonist molecules of the Hh pathway are under consideration for counteracting the EMT and tackling cancer stem cells in smoking-related cancer diseases [118].

### 4.10. Macrophages and CS

Macrophages are known to contribute to CS-induced EMT through the release of cytokines like TGF-β, which are known to directly induce the EMT [119,120]. This study data indicates that the insult-provoking CS induces the recruitment, infiltration, and activation of macrophages in the lungs and these macrophages augment the EMT response to promote fibrosis, inflammation, and cancer growth [121]. Thus, via modulation of macrophage EMT, this may signify a new therapeutic strategy in smoking-related lung disease treatment. Cigarette smoke increases TGF-β signaling in epithelial cells through the TGF-β1 protein and the SMAD pathway and simultaneously increases the level of mesenchymal markers and decreases the level of epithelial markers [1,35,122]. TGF-β signaling also crosstalks with other pathways such as WNT and Notch to enhance the EMT aspect and the stemness and invasiveness properties of CSCs. While that is important, the crosstalk between one pathway and another means that the signaling networks regulating the EMT and CSC properties in cancer are not necessarily simple [7]. A comprehnisve of studies on EMT markers profile in HBECs exposed to cigarette smoke is given in Table 2.

## 5. Strengths and Limitations

This literature review exhibits several notable strengths that contribute to its significance. Firstly, it draws upon recent and up-to-date articles, ensuring the incorporation of the latest findings and advancements in the field. Additionally, this review addresses research gaps concerning normal cell lines, shedding light on aspects of the EMT that were previously understudied. Furthermore, this review emphasizes the importance of investigating the specific role of various constituents present in CS in the EMT process. This recognition underscores the need for a more comprehensive understanding of the complex interactions between CS and cellular mechanisms.

Despite all these studies on CS-induced EMT being conducted using 2D cell culture models, these models bear severe drawbacks (Figure 2). Reconstructed 2D cultures fail to mimic accurately the 3D organization of tissues and the interferences of cell–cell and cell–matrix contacts observed in vivo. On the other hand, 3D cultures seem to provide a physiological environment more similar to the actual tissue architecture for the study of the EMT. This is especially relevant to the investigation of the role of the EMT in the progression of disease in a more physiological context, and also suggests that more attention should be paid to the use of a 3D context for understanding the impact of CS on lung pathology in the future.

Notwithstanding these limitations, this review serves as a valuable resource for researchers, offering valuable insights and guiding them towards fruitful areas of exploration. By highlighting the gaps in current knowledge, it encourages further studies to delve deeper into these areas, thereby advancing our comprehension of the EMT and its implications in the context of CS exposure. Ultimately, this comprehensive examination provides a stepping stone for future investigations to build upon and broaden our understanding of the intricate processes underlying the EMT and its relation to CS-induced effects.

## 6. Conclusions

In conclusion, this review provides comprehensive insight into the molecular analysis and signaling pathways implicated in CS-induced EMT in human bronchial epithelial cells, highlighting key pathways such as WNT/β-catenin, TGF-β/SMAD, hypoxia, oxidative stress, PI3K/Akt, NF-κB, Notch, and Hedgehog. This investigation enriches our understanding of CS-induced EMT and underscores the complex network of signals that drive cancer progression in the context of CS exposure. The insights gained from this review not only consolidate existing knowledge but also pave the way for future research to explore additional complexities of the EMT in the context of CS exposure. The identification of these pathways offers significant potential for therapeutic intervention. Future research should aim to develop targeted therapies to disrupt the EMT process and enhance clinical outcomes in patients with smoking-induced malignancies.

## 7. Methodology

A comprehensive search was conducted in databases including PubMed, Google Scholar, Science Direct, and the Cochrane Library using relevant keywords such as “cigarette smoke”, “epithelial to mesenchymal transition”, “EMT” and “normal human bronchial cell”. The search was focused on recent articles published after 2010. The inclusion criteria included (a) studies that tested the exposure of conventional CS or any of its constituents to human bronchial cell lines; (b) either a long or short duration of exposure; (c) studies reporting any EMT-related characteristics; (d) English-language articles only; and (e) articles from peer-reviewed journals. The exclusion criteria included the following: (a) studies utilizing alternative models other than 2D cell cultures, (b) studies primarily focused on genetic and epigenetic modifications, and (c) literature reviews. Because of the nature of narrative reviews, no formal quality assessment was performed; however, the credibility and relevance of the included studies were considered.

## Figures and Tables

**Table 1:** Significant findings related to smoking and cancer prognosis.

Type of Cancer	Smoking Status	Prognostic Findings	Impact on Survival	Study
Multiple cancers	Current/former smokers	Higher incidence of second primary cancers in smokers.	Significantly reduced overall survival rates.	[6]
Bladder cancer	Current/former smokers	Higher recurrence rates and more aggressive tumor phenotypes in smokers.	Lower disease-free survival and overall survival in smokers.	[6]
Esophageal cancer	Current smokers	Smoking-related esophageal cancer has a higher likelihood of recurrence post-treatment.	Reduced survival and higher recurrence rates post-esophagectomy.	[10]
Lung cancer	Current smokers	Increased risk of second primary lung cancer among smokers.	Reduced overall survival and progression-free survival.	[5]
Non-small cell lung cancer (NSCLC)	Current smokers	Higher incidence of squamous cell carcinoma subtype among smokers.	Lower survival rates in smokers compared to nonsmokers.	[3]
Colorectal cancer	Current/former smokers	Increased pulmonary metastasis in smokers.	Increased mortality due to liver and lung metastasis.	[7]

**Table 2:** Epithelial–mesenchymal transition (EMT) marker profile in human bronchial epithelial cells (HBECs) exposed to cigarette smoke (CS). α-SMA, α-Smooth muscle actin; ZO-1, Zonula occludens-1; FN1, fibronectin 1; PAI-1, Plasminogen Activator Inhibitor -1; MMP-9, Matrix Metallopeptidase 9.

Study	Type of Cell Line	Rate of CS Exposure	Downregulated Epithelial Marker	Upregulated Mesenchymal Marker
[123]	Primary HBE	1.5% CSE for 48 h	E-cadherin	Vimentin, MMP-9
[85]	Primary HBEC	5% CSE for 24 h	E-cadherin ZO-1	Vimentin
[81]	Immortalized 16HBE14o-	40 μmol/L nicotine treated for 72 h	E-cadherin	α-SMA
[100]	Immortalized 16HBE	3% CSE for 48 h	E-cadherin	E-cadherin fibronectin, α-SMA, Collagen 1, Collagen 3
[66]	Immortalized 16-HBE	5, 10 and 20% CSE for 24 h	E-cadherin	N-cadherin, α-SMA, Slug, FN1, Collagen IV
[124]	Immortalized HBE	0.5, 1, and 2% CSE for 24 h	E-cadherin	N-cadherin, Vimentin α -SMA
[65]	Immortalized HBE	1, 2, 4% CSE for 48 h	E-cadherin	N-cadherin, Vimentin, α-SMA, Snail, FoxC1
[55]	Primary HBE	10% CSE for 24 and 48 h	E-cadherin ZO-1	α-SMA, Vimentin
[98]	Immortalized 16HBE	2.5% CSE for 48 h	E-cadherin	α-SMA, Vimentin, MMP-9, MMP-2
[76]	Immortalized 16HBE	2.5, 5% CSE for 72 h, and 5% CSE for 6 h, 12 h, 24 h, 48 h, 72 h	E-cadherin	α-SMA, Vimentin
[77]	Immortalized BEAS-2B	10% CSE for 48 h	E-cadherin	α-SMA
[67]	Primary NHBE Normal human bronchial epithelial cells	2 and 4% CSE for 7 days	E-cadherin ZO-1	N-cadherin, Vimentin
[68]	Immortalized BEAS-2B	5% CSE for 5 days	E-cadherin	Vimentin, N-cadherin, α-SMA
[56]	Immortalized BEAS-2B	1% CSE every 24 h for 48 h	E-cadherin ZO-1 keratin 18	Vimentin, PAI-1, Fibronectin, Snail
[59]	Primary HBEC	6 × 10^−6^ mol/L nicotine for 24 h	E-cadherin	α-SMA, Vimentin, Collagen I, MMP-9

## Data Availability

Not applicable.
